# The Role of TNF-**α** and TNF Superfamily Members in the Pathogenesis of Calcific Aortic Valvular Disease

**DOI:** 10.1155/2013/875363

**Published:** 2013-11-06

**Authors:** Antonella Galeone, Domenico Paparella, Silvia Colucci, Maria Grano, Giacomina Brunetti

**Affiliations:** ^1^Division of Cardiac Surgery, Department of Emergencies and Organ Transplantation (DETO), University of Bari “Aldo Moro”, Italy; ^2^Department of Basic Medical Sciences, Neurosciences and Sense Organs, Section of Human Anatomy and Histology, University of Bari “Aldo Moro”, Piazza Giulio Cesare 11, 70124 Bari, Italy

## Abstract

Calcific aortic valve disease (CAVD) represents a slowly progressive pathologic process associated with major morbidity and mortality. The process is characterized by multiple steps: inflammation, fibrosis, and calcification. Numerous studies focalized on its physiopathology highlighting different “actors” for the multiple “acts.” This paper focuses on the role of the tumor necrosis factor superfamily (TNFSF) members in the pathogenesis of CAVD. In particular, we discuss the clinical and experimental studies providing evidence of the involvement of tumor necrosis factor-alpha (TNF-**α**), receptor activator of nuclear factor-kappa B (NF-**κ**B) ligand (RANKL), its membrane receptor RANK and its decoy receptor osteoprotegerin (OPG), and TNF-related apoptosis-inducing ligand (TRAIL) in valvular calcification.

## 1. Introduction

**Figure 1:**
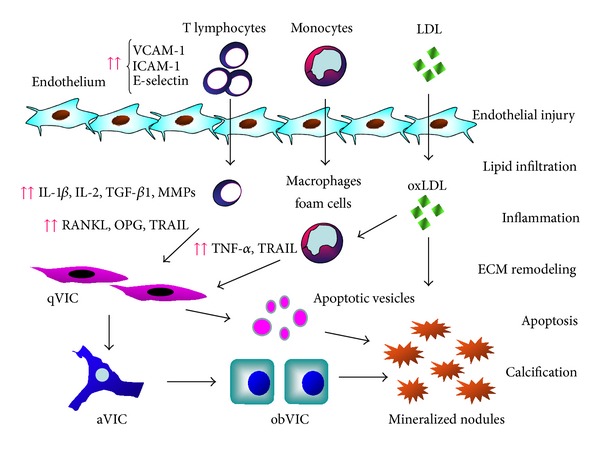
Mechanisms involved in calcific aortic valve disease. An endothelial injury or dysfunction causes increased expression of adhesion molecules, such as VCAM-1, ICAM-1, and E-selectin. Inflammatory cells such as T lymphocytes and monocytes are recruited, and they release cytokines and proteolytic enzymes, which stimulates the activation and differentiation of resident fibroblasts or quiescent valvular interstitial cells (qVICs) into myofibroblasts (activated VICs, aVICs) and osteoblastic VICs (obVICs) with consequent calcification. VICs also undergo apoptosis, and the formation of apoptotic vesicles contributes to calcification. Circulating lipids also enter the valvular interstitial tissue and undergo oxidative modification; the oxidized lipoproteins (oxLDL) are highly cytotoxic and stimulate inflammatory activity and mineralization.

Calcific aortic valve disease (CAVD) represents a slowly progressive pathologic process extending from mild thickening of the aortic valve without obstruction of blood flow, named aortic valve sclerosis, to a severe calcification of valvular leaflets, reduction of valve motion, and obstruction of blood flow, named aortic stenosis (AS) [1]. AS is the most common among heart valve diseases (43.1%) [2]; its prevalence is around 2%, and it increases with age [3–5]. Degenerative etiology is predominant (81.9%) [2]; however, CAVD can no longer be considered a passive process in which the valve degenerates with age in association with calcium accumulation. Instead, CAVD appears to be an actively regulated process including chronic inflammation, lipoprotein deposition, renin-angiotensin system involvement, extracellular matrix (ECM) remodeling, and activation of specific osteogenic signaling pathways and apoptosis, which determine the activation and differentiation of the resident fibroblasts or quiescent valvular interstitial cells (qVICs) into myofibroblasts (activated VICs, aVICs) and osteoblast-like cells (osteoblastic VICs, obVICs) with consequent micro- and macrocalcification [6–8] (Figure 1).

Inflammation is a prominent feature of aortic valve calcification, and it is present in both early and advanced aortic valvular lesions [9, 10]. Histological and immunohistochemical studies showed that early valvular lesions are characterized by a subendothelial thickening of the aortic side of the leaflet with presence of intra- and extracellular lipids and microscopic calcification, as well as interruption of the basement membrane with accumulation of lipids and calcium also in the fibrosa [10]. These lesions are probably consequent to the disruption of the endothelial continuity due to an elevated shear stress, which allows circulating lipids, including low-density lipoprotein (LDL) and lipoprotein (a), to enter the valvular interstitial tissue [11] where they undergo oxidative modification [12]. These oxidized lipoproteins (oxLDL) are highly cytotoxic and capable of stimulating inflammatory activity and mineralization. Valvular endothelial dysfunction or injury also leads to increased expression of adhesion molecules VCAM-1, ICAM-1, and E-selectin and recruitment of inflammatory cells [13]. Normal aortic valves present scattered macrophages and sporadic alpha-actin-positive cells, while T-cells are absent; conversely, early valvular lesions are characterized by an inflammatory infiltrate composed of macrophages (foam cells and nonfoam cells) and T cells and scattered alpha-actin-positive cells [10]. Thus, early lesions of CAVD have some similarities with the atherosclerotic process (lipid accumulation, inflammatory infiltrate, and interruption of the basement membrane) and some differences (presence of early calcification and reduced number of smooth muscle cells). Leukocytes activated in the subendothelium and in the fibrosa induce a chronic inflammation with release of cytokines and enzymes as IL-2 [9], transforming growth factor- (TGF-) *β*1 [7], IL-1*β* [14], TNF-*α* [15], and matrix metalloproteinases (MMPs) [16], which contribute to ECM remodeling, inflammatory activation of myofibroblasts which, in turn, develop an osteoblast-like phenotype, and calcification. Mineralization arises in close proximity to areas of inflammation and has been demonstrated in early [10] as well as advanced lesions [17]. Several features suggest the presence of an active highly regulated process closely resembling developmental bone formation [18, 19]. *In vitro* studies of cultured explants of stenotic valves have identified cells with osteoblastic characteristics that undergo phenotypic differentiation and spontaneous calcification [20]. These osteogenic cells express and produce a variety of regulatory bone matrix proteins including osteopontin (OPN) [21, 22] and bone morphogenetic proteins (BMPs) [17]. The initiation of mineralization (nucleation) may be stimulated by the presence of oxLDL [12, 17] or by the presence of cellular degradation products following apoptosis [8]. 

This paper focuses on the role of the tumor necrosis factor superfamily (TNFSF) members in the pathogenesis of CAVD. The TNFSF is composed of 19 ligands and 29 receptors and plays highly diversified roles in the body [23]. In particular, we discuss the clinical and experimental studies providing evidence of the involvement of tumor necrosis factor-alpha (TNF-*α*), receptor activator of nuclear factor-kappa B (NF-*κ*B) ligand (RANKL), its membrane receptor RANK and its decoy receptor osteoprotegerin (OPG), and TNF-related apoptosis-inducing ligand (TRAIL) in valvular calcification.

## 2. TNF-*α*

Tumor necrosis factor-alpha or TNF-*α* maps to chromosome 6p21.3 and is primarily produced as a 212-amino-acid-long type II transmembrane protein arranged in stable homotrimers [24, 25]. From this membrane-integrated form, the soluble homotrimeric cytokine (sTNF) is released via proteolytic cleavage by the metalloprotease TNF-*α*-converting enzyme (TACE) [26].

TNF-*α* is produced by different kinds of cells, including activated macrophages, monocytes, T-cells, smooth muscle cells, adipocytes, and fibroblasts. The cytokine is involved in acute and/or chronic inflammation. Whereas, in acute inflammation, TNF-*α* protects against bacterial endotoxin, viruses, and parasites, provides increased nutrients for immune cells, and favors a proper host response, in chronic inflammation, TNF-*α* activates pathways responsible for numerous pathological conditions, such as arthritis. In fact, molecules neutralizing it are beneficial in the treatment of diseases. TNF-*α* was aptly named when it was discovered to induce tumor cell apoptosis [27], or programmed cell death. In general, TNF-*α* promotes several cell functions related to immune cell proliferation and adhesion and apoptosis [23, 28].

TNF-*α* can induce biological reactions by either TNF receptor 1 (TNFR1) or TNFR2: the first, which contains a death domain (DD), is highly promiscuous and is expressed on every cell type in the body, whereas the expression of the second receptor is limited to cells of the immune system, endothelial cells, and nerve cells. Each receptor can mediate distinct intracellular signals. In particular, TNF-*α* induces at least 5 different types of signals that include activation of NF-*κ*B, apoptosis pathways, extracellular signal-regulated kinase (ERK), p38 mitogen-activated protein kinase (p38MAPK), and c-Jun N-terminal kinase (JNK). When TNF-*α* binds to TNFR1, it recruits a protein called TNFR-associated death domain (TRADD) through its DD [29]. TRADD then recruits a protein called Fas-associated protein with death domain (FADD), which then sequentially activates caspase-8, caspase-3, and, thus, apoptosis [30]. Alternatively, TNF-*α* can activate mitochondria to sequentially release ROS, cytochrome C, and Bax, leading to activation of caspase-9, caspase-3, and, thus, apoptosis [31].

TNF-*α* has also been shown to activate NF-*κ*B, which, in turn, regulates the expression of proteins associated with cell survival and proliferation [32].   For NF-*κ*B activation, the intracellular domain of TNFR1 is bound by an adaptor protein, TNF receptor-associated death domain (TRADD), which mobilizes additional adaptor protein receptor interacting protein-1 (RIP-1), and TRAF2 [33]. Subsequently, the TRADD-RIP-1-TRAF2 complex is released from TNFR1. The adapter proteins in the complex activate key signaling pathways. RIP-1 recruitment of MAPK extracellular signal-regulated kinase kinase-3 (MEKK3) and TGF-*β*-activated kinase (TAK1) activates the I*κ*B kinase (IKK) complex. The IKK complex phosphorylates I*κ*B*α* that ubiquitinates and degrades I*κ*B*α*. This subsequently releases NF-*κ*B subunits, which translocate into the nucleus and promote gene transcription [34–36]. The proinflammatory effect of TNF-*α* is mediated through NF-*κ*B-regulated proteins, such as IL-6, IL-8, IL-18, chemokines, inducible nitric oxide synthase (iNOS), cyclooxygenase-2 (COX-2), and 5-lipoxygenase (5-LOX), all major mediators of inflammation. Indeed, TNF-*α* can induce expression of TNF-*α* itself through activation of NF-*κ*B [37].

TNF-*α* can also activate cellular proliferation through activation of another transcription factor, activator protein-1 (AP-1) [38], which is activated by TNF-*α* through sequential recruitment of TNFR1, TRADD, TRAF2, MAP/ERK kinase kinase-1 (MEKK1), MAP kinase kinase-7 (MKK7), and JNK. The activation of p38MAPK by TNF-*α* is mediated through TRADD-TRAF2-MKK3. How TNFR2, which lacks a DD, activates cell signaling is much less clear than how TNFR1 activates cell signaling. Since TNFR2 can directly bind to TRAF2, it can activate both NF-*κ*B and MAPK signaling.

Although initially discovered as an anticancer agent, TNF-*α* and its family members have now been linked to an array of pathophysiologies, including cancer, neurologic, pulmonary, autoimmune, metabolic, and cardiovascular diseases [39–47].

*TNF-*α* in CAVD. *Demer first identified that TNF-*α* may participate in vascular calcification, upregulating alkaline phosphate (ALP) activity as a necessary component of calcifying vascular cell mineralization *in vitro* [48]. Thereafter, the role of TNF-*α* in the pathogenesis of aortic valvular calcification has been gradually elucidated; TNF-*α* is a pleiotropic cytokine which induces ECM remodeling [49], cell proliferation and differentiation [15], and calcification [50]. Kaden showed that TNF-*α* is expressed by macrophages in calcific aortic valves and it stimulates *in vitro* proliferation of human valvular myofibroblasts as well as their expression of MMP-1 [49]; normal valves present rare macrophages and low expression of TNF-*α*, MMP-1; conversely, calcific aortic valves present inflammatory infiltrate and colocalized expression of TNF-*α*, MMP-1 [49]. Aortic valve calcification is associated with an osteoblast-like phenotype of local myofibroblasts and is actively regulated by an inflammatory process involving TNF-*α*. Upon stimulation with TNF-*α*, human aortic valve myofibroblasts cultured under mineralizing conditions showed increased formation of calcified, ALP-enriched cell nodules, ALP activity, concentration of the bone-type ALP isoenzyme, and concentration of osteocalcin (OCN), all of which are markers of an osteoblast-like cellular phenotype [15]; by electrophoretic mobility shift assay, DNA binding of the essential osteoblastic transcription factor runx2/cbfa-1 was increased compared to untreated controls [15]. TNF-*α* increases the gene expression of the osteogenic makers ALP and BMP-2 and induces calcification of VICs obtained from the patients with AS [50]; TNF-*α*-induced calcification, ALP activation, and NF-*κ*B and BMP-2 gene expression are inhibited in the presence of inhibitors of NF-*κ*B signalling, showing that TNF-*α* activates the NF-*κ*B signalling pathway and translocates NF-*κ*B p65 subunit into the nucleus for upregulation of the BMP-2 and NF-*κ*B genes [50]. Oxidized lipoproteins have been detected in stenotic aortic valves where they stimulate inflammatory activity [12]; valves with higher oxLDL content had a significantly higher density of inflammatory cells and expression of TNF-*α*, as well as an increased tissue remodeling [51]. Additional experimental evidences support the important role of TNF-*α* in CAVD [52]. IL-1 receptor antagonist-deficient (IL-1Ra^−/−^) mice spontaneously develop AS, and T-cells from IL-1Ra^−/−^ produce much higher levels of TNF-*α* after anti-CD3 antibody stimulation compared to wild-type mice; furthermore, TNF-*α* deficiency completely suppressed AS development in IL-1Ra^−/−^ mice, suggesting that TNF-*α* actively participates in AS development in IL-1Ra^−/−^ mice [52]. Circulating levels of TNF-*α* are elevated in patients with severe AS and correlate with the severity of the hemodynamic pressure overload; moreover, the peripheral TNF-*α* and TNF receptor levels increase in direct relation to deteriorating NYHA functional classification [53]. Circulating TNF-*α* levels reduce progressively, returning to normal 6 months after surgical aortic valve replacement (AVR) [54].

## 3. RANKL/RANK/OPG

The RANKL/RANK/OPG pathway was initially described in the context of bone mass regulation, but now its prominent role in cardiovascular disease is emerging [55].

RANKL is encoded by a single gene at human chromosome 13q14. Alternative splicing of RANKL mRNA allows expression as a type II transmembrane glycoprotein of either 316 or 270 amino acids or as a soluble ligand of 243 amino acids [56, 57]. In addition, RANKL can be released from its membrane-bound state by metalloproteinases [58, 59]. RANKL is expressed by activated CD4+ and CD8+ T lymphocytes, double-negative thymocytes, immature B lymphocytes, osteoblasts, osteocytes, bone marrow stroma, vascular endothelia, developing lymph node anlage, and developing breast epithelia [56, 60–64]. RANKL acts following the binding with RANK which plays a crucial role in bone homeostasis and lymphoid tissue organization [64–67]. In particular, RANKL is the master cytokine driving osteoclast differentiation. The strongest evidence for the role of RANKL during osteoclastogenesis came from gene inactivation in murine models [56, 67–69], leading to osteoclast-poor osteopetrosis already present at birth. At 1 month of age, RANKL^−/−^ mice were severely growth retarded due to poor nutrition secondary to lack of tooth eruption and displayed shortened long bones with club-shaped ends, thinning of the calvariae, generalized increase in bone density with very little marrow space, marked chondrodysplasia with thick, irregular growth plates, and relative increase in hypertrophic chondrocytes. Moreover, RANKL^−/−^ mice displayed defects in the immunological compartment: reduced thymus size, spleen enlargement, complete lack of lymph nodes, and smaller Peyer's patches [56, 70, 71].

RANK is a type I transmembrane glycoprotein encoded on human chromosome 18q22.1 and is expressed on the surface of osteoclasts and osteoclast precursors as well as bone-marrow-derived dendritic cells, activated T-cells, vascular endothelia, chondrocytes, bone marrow fibroblasts, and mammary gland epithelia. Each RANKL trimer engages three molecules of RANK. Trimerization triggers a conformational change in the cytoplasmic domain of RANK that allows recruitment of TNFR-associated factors (TRAFs). In particular, TRAF2 and TRAF6 are the most critical for RANK signalling [72–74]. TRAF2 mediates activation of AP-1 in concert with ASK1 [75, 76]. TRAF6 makes complexes with c-Src and c-Cbl to activate PI3K, leading to PKB activation and cytoskeletal reorganization [77–79]. Moreover, TRAF6 activates microphthalmia transcription factor (MITF) by activating the p38 microtubule-associated protein kinase pathway through TAB2 and TAK1 [80].

OPG, encoded by a single gene on chromosome 8q24, is a soluble, 110 kDa, disulfide-linked, homodimeric glycoprotein that functions as a decoy receptor for RANKL. Thus, OPG modulates osteoclast formation by inhibiting RANK activation [62]. OPG also can bind the TNFSF member TRAIL, and it has been found that OPG inhibits TRAIL-induced apoptosis of Jurkat, LNCaP cells in culture and of osteoclast, and malignant plasma cells in multiple myeloma [81–85]. OPG mRNA has been detected in B cells, bone-marrow-derived and follicular dendritic cells, vascular endothelia, VSMCs, heart, lung, kidney, bone, stomach, intestine, placenta, liver, thyroid, skin, spinal cord, and brain [86–93].

Transgenic mice expressing OPG exhibited increased bone density, which was explained histologically by a marked decrease in osteoclast number that was presumably due to reduced osteoclast formation [87]. In animals expressing high levels of OPG, the bones were virtually solid, lacking a visible marrow cavity and with nonresorbed cartilage remnants visible histologically within trabeculae [87]. By contrast, mice deficient in OPG developed osteopaenia at an early age owing to increased osteoclast activity, thereby underscoring a physiological role for OPG in the maintenance of normal bone mass [94]. In addition, OPG^−/−^ mice develop arterial calcification, suggesting that OPG plays a role in the maintenance of VSMCs homeostasis [94]. OPG could act as an inhibitor of vascular calcification, whereas RANKL promotes extracellular mineralization of cultured VSMCs via a BMP-4-dependent mechanism [95].

*RANKL/RANK/OPG in CAVD*. Kaden et al. first showed by immunohistochemistry that RANKL and OPG are differentially expressed in calcific AS. RANKL is present in aortic valves from patients with AS, while it is not expressed at relevant levels in normal valves; conversely, OPG expression is marked in normal valves but significantly lower in AS. Additionally, areas containing focal calcification exhibit significantly less OPG-positive cells as compared to noncalcified regions [96]. Further studies support the concept that RANKL/RANK/OPG system exhibits a differential profile throughout the progression of the disease. In particular, the percentage of cells labeled by OPG, RANK, and NF-*κ*B is increased in sclerotic valves compared with stenotic valves, whereas the frequency of RANKL is higher in stenotic compared to sclerotic valves. As a consequence, the OPG/RANKL ratio is decreased in stenotic compared to sclerotic valves [97]. Other studies showed that there is a progressive increase in the gene expression of OPN, bone sialoprotein II, and OPG in the clinical continuum from healthy valves to heavily calcified ones; conversely, BMP-2 and -4 gene expression is significantly decreased in calcified valves suggesting that the expression of pro- and anticalcific noncollagenous bone-associated matrix proteins is altered during the disease continuum and that this imbalance may contribute to the pathology of CAVD [98]. In cultured human aortic valve myofibroblasts, stimulation with RANKL leads to a significant rise in matrix calcification, nodule formation, ALP activity, expression of the bone-type isoenzyme of ALP, and expression of OCN; moreover, RANKL increased DNA binding of the essential osteoblast transcription factor runx2/cbfa-1 [96]. RANKL is also involved in connective tissue remodeling; the addition of RANKL to the culture medium of human aortic valve myofibroblasts induces cell proliferation and MMP expression and activation as compared to medium alone [99]. Experimental studies showed that exogenous OPG protects aortic valve function in hypercholesterolemic *Ldlr* ^−/−^ *Apob* ^100/100^ mice, which are prone to develop calcific AS. OPG profoundly attenuates valve calcification by inhibition of osteogenic transformation, but it does not prevent valve fibrosis or lipid deposition; in particular, OPG strongly suppresses levels of osterix, OCN, and monocyte-chemoattractant protein-1 [100]. In patients undergoing AVR surgery for AS, plasma levels of RANKL, runx2/cbfa1, and tartrate-resistant acid phosphatase (TRAP) exhibited a significant correlation to the severity of AS; in the same patients, mRNA levels of RANKL, RANK, and TRAP are significantly elevated in calcified parts of the valves compared to normal and thickened parts of the same valves obtained at time of surgery [101]. In patients with symptomatic AS, the levels of circulating OPG are poorly correlated with the degree of AS, but they are significantly associated with impaired cardiac function and all-cause mortality [102]. In patients with severe AS scheduled for AVR, preoperative circulating OPG levels are associated with left ventricular and left atrial remodeling; moreover, increasing OPG levels are associated with a poor postoperative outcome after surgery [103]. Interestingly, circulating OPG levels significantly change after surgical AVR, but they remain without any significant differences after transcatheter aortic valve implantation [104]. 

## 4. TRAIL

Tumor necrosis factor- (TNF-) related apoptosis-inducing ligand (TRAIL/Apo2L), located on chromosome 3, as a member of the TNF superfamily of proteins, is expressed as a type II transmembrane protein. Cleavage of its C-terminal part (extracellular domain) allows for a soluble form of TRAIL [105–107].

TRAIL is mostly expressed by cells of the immune system where it was shown to play a role in the homeostasis of certain T-cells and in NK and T-cell-mediated killing of virally and oncogenically transformed cells [108–110]. TRAIL forms homotrimers that bind receptors present on the cell surface. This trimerization enhances the biological activity of TRAIL as compared to monomeric forms of TRAIL [106]. To date, TRAIL has been shown to interact with five receptors, including the death receptors DR4/TRAIL-R1/TNFRSF10A [111] and DR5/TRAIL-R2/TNFRSF10B [112–115] as well as the decoy receptors DcR1/TRAIL-R3/TNFRSF10C [112, 113] and DcR2/TRAIL-R4/TNFRSF10D [114]. In addition to these four membrane-bound receptors, TRAIL is also able to bind to OPG [81]. DR4 and DR5 are type I transmembrane proteins that contain a death domain in their cytoplasmic domain that can bind to other death domains. Upon binding of TRAIL trimer, DR4 and DR5 are oligomerized and can then transduce the apoptotic signal. Inversely, DcR1 and DcR2 can transduce an apoptotic signal. Indeed, DcR1 is bound to the membrane exclusively through a glycosylphosphatidylinositol (GPI) anchor, hence, lacking the entire cytoplasmic domain, and DcR2 contains a truncated and nonfunctional death domain. Hence, even though TRAIL binds to the decoy receptors, the apoptotic pathway cannot be engaged. This competition for the binding to TRAIL was first thought to be the mechanism behind the resistance of certain tumor cells to TRAIL-mediated apoptosis. TRAIL binding to DR4 and DR5 induces recruitment of the adapter molecules Fas-associated death domain (FADD) that leads to direct activation of the caspase cascade. This activation is accomplished by recruitment of caspase-8, followed by its proteolytic activation. Once activated, caspase-8 can proteolytically cleave the BH3-interacting death domain agonist (Bid), a proapoptotic member of the Bcl-2 family proteins, leading to the formation of a truncated Bid form (tBid) that, in turn, activates the mitochondrial apoptotic pathway [115–117]. Alternatively, the activated initiator caspase-8/-10, in turn, targets the effector caspase-3 for proteolytic cleavage which, once activated, cleaves other caspases as well as numerous regulatory and structural proteins [118, 119], resulting in the appearance of the hallmarks of apoptosis such as membrane blebbing, internucleosomal DNA fragmentation, and nuclear shrinkage [120].

TRAIL firstly received considerable attention as a molecule showing the ability to induce apoptosis in a wide variety of neoplastic cells [121]. However, many normal cells, such as thymocytes [121], neural cells [122], hepatocytes [123], osteoclasts [124–126], osteoblasts [127–129], VSMCs [130], and VICs [8], are sensitive to TRAIL-induced apoptosis.

*TRAIL in CAVD*. VICs sensitivity to TRAIL apoptotic effect is of paramount importance because apoptosis has been shown to be an initiator of vascular calcification in *in vitro* studies [131]; increased apoptosis precedes calcification in VSMC cultures, and apoptotic bodies may act as nucleating structures for calcium crystal formation [131]. Previous studies focused on the role of apoptosis in the pathogenesis of CAVD [7, 132, 133].

TGF-*β*1 is present in human calcific aortic stenotic cusps and promotes calcification of cultured sheep aortic VICs (SAVICs) through mechanisms involving apoptosis [7]; in fact, the administration of an apoptosis inhibitor to SAVICs cultured in an osteogenic environment results in a significant decrease in nodules calcification, thereby demonstrating that a certain level of apoptosis is necessary for the calcification of nodules in these cultures [7]. TRAIL has been detected in atherosclerotic lesions [134], and TRAIL-expressing T-cells induce apoptosis of VSMCs in the atherosclerotic plaque [130]. TRAIL is expressed in human calcified aortic valves but not in normal ones, and it is mainly produced by T-cell and macrophages. Moreover, serum levels of TRAIL are significantly elevated in patients with CAVD compared to normal subjects [8]. VICs derived from calcific and noncalcific aortic valves express both death and decoy TRAIL receptors; in particular, VICs derived from calcific valves show significantly higher gene and protein levels of DR4, DR5, DcR1, and DcR2 compared to VICs derived from noncalcific valves [8]. Additionally, VICs derived from calcific valves express significantly higher levels of runx2 compared to VICs from noncalcific valves; thus, the osteoblast-like phenotype is also associated with a higher expression of all TRAIL receptors [8]. The expression of TRAIL receptors in human VICs is associated with the sensitivity to TRAIL-mediated apoptosis involving caspase-3 activation [8]. VICs cultured in an osteogenic medium express higher mRNA levels of runx2 and OCN, together with the increase of DR4 levels compared to medium alone [8]; moreover, the addition of TRAIL to the osteogenic medium leads to a significant increase of mineralized matrix nodule deposition [8]. Taken together, all of these results suggest an active role of TRAIL-induced apoptosis in the pathogenesis of CAVD.

## 5. Conclusions

Although, to date, no medical therapeutic options are able to prevent or reduce the progression of CAVD and the only treatment for severe AS is surgical aortic valve replacement (AVR), the understanding of the underlying pathogenic mechanisms of the disease is mandatory to identify promising therapeutic targets. It is known that the recently available biologic drugs neutralizing RANKL and TNF-*α*, key cytokines in CAVD pathogenesis, are having a great success in the treatment of osteoporosis and arthritis, respectively [135, 136]. Thus, it could be that in the future these molecules could be useful in CAVD treatment/prevention, also because a strong association has been demonstrated between arterial and valvular calcification and osteoporotic bone remodelling [137].
